# Guideline for the application of heart rate and heart rate variability in occupational medicine and occupational health science

**DOI:** 10.1186/s12995-024-00414-9

**Published:** 2024-05-13

**Authors:** Stefan Sammito, Beatrice Thielmann, Andre Klussmann, Andreas Deußen, Klaus-Michael Braumann, Irina Böckelmann

**Affiliations:** 1 Department of Occupational Medicine, Medical Faculty, Otto-von-Guericke University Magdeburg, Magdeburg, Germany; 2 German Air Force Centre of Aerospace Medicine, Experimental Aerospace Medicine Research, Flughafenstraße 1, Cologne, 51147 Germany; 3 Competence Centre Health (CCG), Department Health Sciences, University of Applied Sciences (HAW) Hamburg, Hamburg, Germany; 4 Department of Physiology, Medical Faculty, TU Dresden, Dresden, Germany; 5 Department of Sport Medicine, Universität Hamburg, Hamburg, Germany

**Keywords:** Autonomous nervous system, Sympathetic nervous system, Parasympathetic nervous system, Stress, Strain

## Abstract

This updated guideline replaces the “Guideline for the application of heart rate and heart rate variability in occupational medicine and occupational health science” first published in 2014. Based on the older version of the guideline, the authors have reviewed and evaluated the findings on the use of heart rate (HR) and heart rate variability (HRV) that have been published in the meantime and incorporated them into a new version of this guideline.

This guideline was developed for application in clinical practice and research purposes in the fields of occupational medicine and occupational science to complement evaluation procedures with respect to exposure and risk assessment at the workplace by the use of objective physiological workload indicators. In addition, HRV is also suitable for assessing the state of health and for monitoring the progress of illnesses and preventive medical measures. It gives an overview of factors influencing the regulation of the HR and HRV at rest and during work. It further illustrates methods for measuring and analyzing these parameters under standardized laboratory and real workload conditions, areas of application as well as the quality control procedures to be followed during the recording and evaluation of HR and HRV.

**Supplementary Information:**

The online version contains supplementary material available at 10.1186/s12995-024-00414-9.

## Preliminary remarks

In addition to individual minor editorial and content-related changes, the updated guideline contains major changes in the following areas:

- Statement on the usability of mobile wearables (Section: Technical possibilities and requirements),
- Discriminatory power between HRV and pulse wave variability (Section: Technical possibilities and requirements),
- Addition of innovations in the field of measurement technology since 2014 (Table 1),
- Updating the overview table of HRV parameters (Appendix 1),
- Updating the evidence on factors influencing HRV and restructuring (Table 2),
- Additions to the usability of the HR under dynamic loads (Section: Heart rate),
- Complete revision of the chapter on HRV reference values (Section: Heart rate variability),
- Update on the use of HRV in the prognosis of diseases (Section: Application for risk stratification of diseases).

## Introduction

The HR provides information about the strain of the cardiovascular system in response to physical and mental workload. The HRV gives additional information regarding the dynamics and mechanisms of cardiovascular regulation [1]. Both physiological parameters have been established for the use in inpatient and outpatient care (e.g. cardiology, intensive care, endocrinology, neurology, occupational medicine, sports medicine, obstetrics) as well as medicine and scientific research (occupational physiology, exercise physiology, occupational science, sport science, psychology and pharmacology) for many years because of their non-invasive data acquisition and comfortable methods of, analysis.

## Definitions

In medical assessment, a distinction is required between the HR (measured centrally) and the pulse rate (measured peripherally). A difference can occur, for example, in certain forms of cardiac arrhythmia as a pulse deficit.

HR is defined as the number of beats or contractions of the heart per minute. It can also be calculated as a ratio of 60,000 and the average NN interval^1^ in milliseconds. The HR is a measure of the individual workload response of the cardiovascular system and is affected by various factors (see Section: Factors influencing the individual HR and HRV). HR should be distinguished from the pulse rate, which is defined as the number of pulsations per minutes palpated at the periphery, e.g. at the wrist or at the neck. A difference between HR and pulse rate may occur in certain types of cardiac arrhythmias where some contractions of the heart do not produce a palpable pulse at the periphery. A difference between the HR and the pulse rate is called pulse deficit.

In adults, the resting HR (HRrest) is usually between 60 and 80 beats/min (bpm). In people with endurance training, values well below 50 bpm may be obtained at rest

The HR_rest_ typically varies between 60 and 80 bpm in adults. It is usually higher in children i.e. up to 120 bpm [2]. In endurance trained adults, the HR_rest_ is often below 50 bpm.

The maximum achievable HR varies greatly between individuals and depends on age and biological sex, among other factors. It is recommended to determine it individually as part of ergometric exercise during general dynamic muscle work. The use of formulae to estimate the maximum heart rate (HR_max_) should be used with great caution due to the very wide variation

The HR reaches a maximum during physical exertion. The maximum value differs between individuals and decreases with age. The most commonly used empirical formula for estimating the HR_max_ is [3]:

$${\text{HR}}_{\max} = 220 - \text{age}$$

However, this formula underestimates the HR_max_ in individuals > 40 years of age [4]. Based on a meta-analysis and their own examinations, Tanaka et al. [4] calculated a regression formula to estimate age-dependent HR_max_ by:

$${\text{HR}}_{\max} = 208 - 0.7\times \text{age}$$

in which sex-related differences have not been considered [4, 5]. The high inter-individual heterogeneity of the HR_max_ as a function of age is confirmed by clinical studies for women [6] and men [7]. The determination of the individual HR_max_ requires maximum physical exertion under conditions of dynamic muscle activity of a larger muscle mass, e.g. a cardiac stress test using treadmill or bicycle ergometry [8]. Depending on the specificity of the subjects, usually other instruments like the arm crank ergometer may be used as well [9].

The recovery HR (HRrecovery), the working HR (HRwork) and the integral of the HRrecoveryare available for further assessment of the performance of the cardiovascular system

In the field of exercise physiology, the HR following a maximal exercise test is frequently taken as an indicator of the fitness level of a subject. The value is measured one minute^2^ after the cessation of a maximal exercise test. It reflects the rapid regulative phase of recovery and is called the HR_recovery_.

In the fields of occupational medicine and occupational health science, the *HR during work* 3 (HR_work_) is taken into consideration while analysing the respective activity, e.g., evaluation of physical work. The HR_work_ is defined as the difference between HR_rest_ and the value measured during physical work [10–12]. HR_work_ is also known as *net HR* (HR_net_) [13]. HR_net_ correlates better with the physical exertion than the HR, provided a resting phase without physical or emotional stress of at least five minutes (ideally, fifteen minutes) before starting the work is maintained to assess a valid baseline HR. This is a necessary requirement while carrying out tests in the laboratory, whereas in cases of measurements at real workplaces, it could be difficult to achieve these conditions before the working shift. Under circumstances of unreliable and not representative HR_rest_ measurements, absolute HR might better reflect the intensity of the workload during physical exertion than HR_work_. Alternatively, the *reference HR (HR*_*reference*_ *)* can also be determined for light dynamic work (see Section Heart rate at rest).

In addition, the individual physical exertion is also frequently described by calculating the *summated recovery HR* as a measure of the fatigue and recovery [14]. For this procedure, all heart beats during the recovery phase are summed until HR reaches the baseline level (e.g., HR_rest_) (Calculation of the integral of the heartbeats above HR_rest_ over the time from end of exercise to normalisation of HR).

HRV is based on a mathematical analysis of a time series of consecutive heart actions - the so-called NN intervals.

**Fig. 1:**
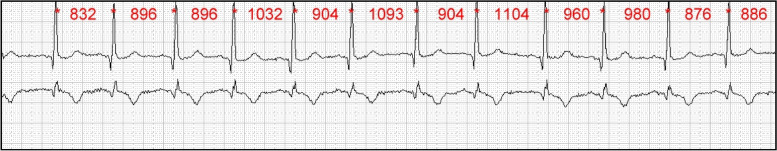
Principle of determining the NN intervals from the ECG as a measure of the distance between two R-spikes

The term *HRV* comprises a number of mathematically calculated parameters, which characterise the variance, rhythm or complexity of a time series of consecutive heart beats – the so-called NN interval. Because of robustness and reliability issues the R-wave is usually used as a sign of electrical heart activation during automatic detection (Fig. 1). A detailed list of the frequently used HRV parameters can be found in Section: Analytical methods and parameters of HRV.

## Physiological mechanisms

### Physiological mechanisms of HR

The autonomic modulation of HR by sympathetic cardiac nerves and the vagus nerve (parasympathetic) is primarily mediated via the sinus node.

During the resting phase, the frequency of the heartbeat is triggered by the primary impulse generating tissue (pacemaker), the sino-atrial node (SA-node). The frequency of the non-innervated sinus node is stated differently in the literature. In the short term after heart transplantation, in which the transplant is denervated by the surgery, the HR is higher than the normal physiological HR, which is 60 to 80 bpm [15]. For a longer period of time after transplantation, it changes back towards normal physiological HR, probably due to partial reinnervation. After transplantation, however, the beta-adrenergic activation of the sinus node cells by circulating adrenaline from the adrenal medulla continues, which may contribute to the increased HR_rest_. All downstream pacemaker tissues also capable of spontaneous depolarisation (AV node, bundle of His, Purkinje fibres) exhibit lower activation frequencies. The autonomic modulation of HR by the sympathetic and the parasympathetic (on the heart singularly influenced by the vagus nerve) is primarily mediated by the SA-node. This two-way control of the autonomic nervous system (ANS) was demonstrated in blocking experiments of the adrenergic (beta-blockers) and muscarinic-cholinergic (atropine) receptors [16–18].

### Physiological mechanisms of HRV

The HR is subject to physiological variability even during constant stress, which reflects the interaction of the sympathetic nervous system (SNS) and vagus nerve (as part of the parasympathetic nervous system [PNS]), among other factors.

Even under constant physical exertion, HR shows a physiological variability, which predominantly reflects the interplay in the ANS between the SNS and the N. vagus (as part of the PNS). The sympathetic part of the ANS leads to a reduced HRV through the release of adrenaline and noradrenaline, while the parasympathetic (vagal) part leads to an increase in HRV through the release of acetylcholine [19].

At rest and during mild exertion, the parasympathetic (vagal) control outweighs the sympathetic effect. This leads to an increased variability of the heartbeats: the difference in the gap between two consecutive heartbeats increases.

The HRV analysis is used particularly for the differential evaluation of the interplay between the sympathetic and the PNS under various conditions. Therefore, the quantification of the autonomic activity is carried out by analysing the periodic fluctuations of the heartbeat. Rapid changes in the HR with a cycle length of about 2–7 s are closely associated with breathing (Respiratory Sinus Arrhythmia [RSA]). These high-frequency fluctuations are modulated almost exclusively by the parasympathetic branch of the ANS (vagus nerve); whereas the slow fluctuations (cycle length of about 10 s) are modulated by the efferent nerve fibers of both parts of the ANS [20]. However, for the interpretation of HR and HRV, it must be taken into consideration that both parameters reflect the net effect of autonomic cardiac efferent nerve activity but also other modulating factors like humoral and mechanical influences during physical exertion, heat and other environmental factors. In the case of temperature changes, the effects are mediated on the one hand via the modulation of the ANS and on the other hand directly via temperature effects on the sinoatrial node cells of the heart.

The vagal resting tone is higher the better the heart is adapted to cope with high physical stress, which is why trained people (e.g. endurance athletes) generally have a higher HRV in addition to a lower HR_rest_. In addition to changes in the activity of autonomic efferent nerve fibers, endurance training also leads to changes in the expression of ion channel proteins and membrane transport proteins [21], which has an additional lowering effect on HRV.

## Determination of the NN intervals for the calculation of HR and HRV

### Technical possibilities and requirements

Different measuring systems are available for recording heart actions. Their measurement accuracy for a subsequent HRV analysis varies. It is recommended to use an ECG-based measurement for this purpose. The devices should measure non-invasively at a high sampling rate (ideally 1,000 Hz, minimum 250 Hz), be mechanically robust and non-reactive.

Several methods are available to record the interbeat intervals: stationary ECG instrument – which is more suitable for laboratory studies or intensive care units – and mobile measurement techniques that are convenient in field studies. The mobile measuring systems include 24-hour ECG devices, chest strap systems with direct storage or storage on an external data module (e.g. in a separate heart rate monitor) as well as a number of mobile measuring systems that have become available on the market in recent years (e.g. watches without a chest strap, systems with measurements in the ear canal, etc.). What all measuring systems have in common is that they record the NN intervals with different levels of measurement accuracy and deviations can therefore occur in the subsequent HRV analysis [22, 23].

Pulse oximeters can also determine the distance between two pulse waves. However, these differ from the NN intervals measured on the heart using electrodes. A systematic review [24] showed that only in young, healthy subjects under resting conditions are there acceptable correspondences between NN intervals and pulse NN intervals, but HRV parameters based on the pulse NN intervals are sometimes significantly increased (e.g. in HF). It is therefore important to make a strict distinction between HRV and the so-called pulse rate variability (PRV).

For HRV analysis, a so-called “beat-to-beat recording” with assessment of all cardiac actions and a high sampling rate (ideally 1,000 Hz, minimum 250 Hz [25]) is gold standard in order to record the distances between individual cardiac actions with high temporal accuracy.

In addition, the instruments should fulfill the following requirements:

- non-invasive,
- mechanically robust (for examinations at workplaces, which involve heavy physical work or difficult environmental factors like heat, cold and wet conditions) and.
- non-interfering (the method itself should not influence the results in any way).

The advantages and disadvantages of the different measurement systems are given in Table 1.

**Table 1:** Advantages and disadvantages of the different measurement systems

	Advantages	Disadvantages
Stationary (24-hour) ECG	• ECG recording • non-invasive • visual monitoring of R-wave detection • medical device according to the Medical Devices Act	• not portable, suitable only for laboratory examinations and intensive care units • bothersome cable
Portable (24-hour) ECG	• portable, small devices • suitable for laboratory and field studies • ECG recording • non-invasive • visual monitoring of R-wave detection • medical device according to the Medical Devices Act	• bothersome cable
Chest strap systems with storage in a separate heart rate monitor	• portable, small devices • high freedom from reaction • non-invasive	• no ECG recording • interference with data transmission (because of power lines, vehicles, etc.) • not a medical device according to the Medical Devices Act
Chest strap systems with storage directly in the chest strap	• portable, small devices • high freedom from reaction • non-invasive	• no ECG recording • not a medical device according to the Medical Devices Act
Watch systems without a chest strap and with sensors to record the pulse wave	• portable, small devices • high freedom from reaction • non-invasive	• no ECG recording • formally no NN interval recording • not suitable for measuring HRV • not a medical device according to the Medical Devices Act
Measurement in the ear canal	• portable, small devices • high freedom from reaction • non-invasive	• no ECG recording • formally no NN interval recording • not suitable for measuring HRV • not a medical device according to the Medical Devices Act
Measurement using ear clips based on a pulse oximeter	• portable, small devices • high freedom from reaction • non-invasive	• no ECG recording • formally no NN interval recording • not suitable for measuring HRV • not a medical device according to the Medical Devices Act

Devices according to the Medical Devices Act are specifically for diagnostic or therapeutic purposes and are intended by the manufacturer for use on humans

### Electrodes

It is recommended that the electrodes be adequately prepared for optimal measurement results.

The following should be done to avoid errors during measurement:

- adhesive electrodes should be used so that they do not lose contact with the skin even after longer periods of recording (e.g. 24 h) and in cases of sweating,
- the electrodes on the chest belt (contact points) should be moistened,
- the chest belt should fit firmly and.
- a textile strap should be preferred, because it can adapt itself optimally to the individuals upper body.

### Preparation of the skin

It is recommended that the skin be adequately prepared for optimal measurement results. This includes, among other things, reducing the oil film on the skin and, if necessary, removing existing (chest) hair.

The skin should be prepared carefully in order to obtain optimal results of measurements, especially if long-term recordings (24 h) are carried out. In cases of skin-electrode contact with high impedance, the quality of the recording decreases and the probability of the appearance of artifacts are high.

The main objective of preparing the skin is to remove the natural oil film of the skin. This reduces the contact resistance between the skin and electrodes and enables a better adherence of the electrodes. The contact points on the skin for the electrodes are first cleaned with a dermatologically safe, degreasing solution (e.g. alcohol solution). However, any damage or injury to the skin has to be avoided. If chest hair grows, it may be necessary to carefully remove the hair from the corresponding adhesive areas before attaching the adhesive electrodes. An additional fixing of the electrodes and the cables is useful for long-term recordings or other conditions.

### Lead choice and electrode positioning

It is recommended to select the ECG lead with the largest amplitude of the R wave of the QRS complex.

The ECG leads must be chosen based on the largest amplitude of the R-wave of the QRS complex (see Fig. 1). In principle, recordings from a single lead are sufficient. If possible, multiple leads should be used to enable a reliable correction of artifacts.

During the automatic determination of the NN interval, it should be ensured that R-wave detection is consistently based on the same lead. Changing the lead during the same recording can lead to an artificially generated increase in the HRV. While the point of time at which the QRS complex begins is almost identical in most of the leads, the fiducial point (R-wave), which serves as the basis for determining the NN interval, can significantly vary between the different leads [26, 27].

The positioning of the electrodes influences the quality of the recordings. If electrodes are not positioned appropriately, recording quality might suffer, resulting in an accumulation of artifacts. The intercostal spaces are suitable areas for positioning the electrodes. Within these spaces, flat and even areas of the skin should be selected (e.g. positioning above dermal naevi should be avoided).

### Quality assurance while determining the HR

When recording the HR, quality assurance requires the determination of HR_rest_, artifact control, the highest possible sampling rate and the consideration of possible influencing factors.

The following aspects should be taken into consideration for the purpose of quality assurance:

- the determination of HR_rest_ 4 before the beginning of the exertion as physiological baseline for the evaluation (see Section: Heart rate at rest),
- checking for artifacts and, if possible, removal of artifacts (e.g. by visually checking the data during analysis, automatic methods for correcting artifacts),
- a high sampling rate (see above),
- the possible influencing factors depending on the case (see Table 2) and.

**Table 2:** Factors influencing HR and HRV, sorted according to the four main areas depicted in Fig. 4, sorted alphabetically within the main area

Influencing factor	Effect on resting heart rate (HR_rest_)	Effect on heart rate variability (HRV)
**Non-influenceable physiological factors**		
Age	HR_rest_ [28] and HR_max_ [8] normally decrease with increase in age	HRV increases sharply in the first year of life, then increases until the age of 15 [29], is highest in young adulthood and falls non-linearly with age [28–46].
Circadian rhythm/time of the day	The HR follows a circadian rhythm, with a fall of HR at night [47].	The HR follows a circadian rhythm, but the HRV is decreased at night due to the predominance of the parasympathetic activity and reduced during the day because of the predominance of the sympathetic activity [48].
Genetic		HRV appears to vary between members of different ethnic backgrounds [49].
Pregnancy	During pregnancy there is usually an increase in HR [50].	During pregnancy, a reduction in HRV usually occurs as the pregnancy progresses [50] and is lowest in the 2nd trimester [51, 52].
Biological sex	The HR is normally higher in women than in men [53].	Most of the studies showed a higher parasympathetic activity in women as compared to men [31, 32, 54–59], which however showed a narrower difference after the age of 50 [33–35]. Some of the studies showed a higher baseline sympathetic activity in women [36, 37, 60, 61].
**Diseases**		
Cancer diseases		The influence of **breast cancer** on HRV is unclear [62].
Cardiovascular diseases	**Cardiac insufficiency** leads to a raised HR [63] and unrestricted maximum HR.	**Cardiac insufficiency** generally leads to a reduction in the HRV [5, 64–67].
		With **hypertension**, HRV is usually reduced [68, 69].
	In patients with previous **myocardial infarction**, the activation of the sympathetic nervous system often leads to an increase in the HR, which is important for the prognosis [70–75].	HRV is usually reduced in **coronary heart disease** (CHD) with and without angina pectoris and after myocardial infarction [76, 77].
Chronic obstructive pulmonary disease (COPD)		With COPD, HRV is usually reduced [78, 79].
Chronic renal insufficiency		In chronic kidney failure, HRV is usually reduced [78].
Duchenne muscular dystrophy		HRV is usually significantly reduced in the early stages of **Duchenne muscular dystrophy** and in manifest disease [80].
Headaches, regular		**Regular headaches** are usually associated with reduced HRV [81, 82].
Metabolic disorders	**Diabetes mellitus** is often associated with increased sympathetic activity and hence a raised HR [83].	The HRV is often reduced in patients with **diabetes mellitus** [58, 84–87], however, a correlation between the value of the HRV and the duration of the diabetes exists especially in cases of badly controlled diabetes [88]. The reduction is due to peripheral neuropathy due to microcirculation disorders [89].
		A **metabolic syndrome** often leads to a reduction of the HRV [88, 90–96], this is particularly evident in women [97].
Pain		In **chronic pain**, HRV is usually reduced [98].
Psychiatric disorders	Patients with **anxiety disorders** and **panic attacks** usually have an increased HR [99].	Patients with **anxiety disorders** [38, 100–103] and **panic attacks** [99, 101] usually show a reduction in the HRV.
	Patients with **anorexia nervosa** usually have a reduced HR [104].	HRV is usually reduced in patients with **anorexia nervosa** [102].
		HRV is usually reduced in patients with **bulimia nervosa** [105].
		**Posttraumatic stress disorder** often leads to a reduced HRV [106].
	A **major depression** often leads to an increase in HR [107–109].	A (**major) depression** often leads to a decrease in HRV [38, 108, 110–114].
		In **epilepsy**, HRV is usually reduced [115].
		In **borderline personality disorder**, HRV is usually reduced [116].
		In **bipolar disorder** [38, 113, 117, 118]./ **schizophrenia** [119], HRV is usually reduced.
		In the case of **substance addiction** [38], HRV is usually reduced.
Rheumatoid arthritis		Based on a systematic literature search, HRV does not currently appear to be changed in the presence of **rheumatoid arthritis** [120].
Sleep disorders		A reduction in HRV in the presence of **sleep disorders** is currently not supported by the scientific literature [121]. Something similar can be found in untreated **obstructive sleep apnea syndrome**.
Stroke		A **stroke** is usually associated with reduced HRV [122].
**Influenceable lifestyle factors**		
Alcohol consumption		With acute alcohol consumption, HRV is usually reduced [123]. Low, constant alcohol consumption with an alcohol content of one standard drink for women or two standard drinks for men usually leads to a short-term but no long-term change in HRV or an increased HRV, while chronic alcohol abuse leads to a reduction of HRV [123, 124].
Body fat/body weight	Increased body mass index (BMI) generally leads to a raised HR [125], which can be partly explained by the stimulating effect of leptin on central sympathetic neurons [126, 127].	Increased body mass index (BMI) and increased mass of body fat often cause a fall in the HRV [128].
Fitness activities, performance capacity, sports		High-intensity interval training (HIIT) generally increases HRV, which has been shown particularly in healthy subjects and patients with metabolic syndrome [129]. High-intensity training and competition series, on the other hand, can lead to reduced HRV [130, 131].
		During strength training, there is usually no change in HRV in healthy people, while strength training is usually associated with an increase in HRV in subjects with chronic illnesses [132].
	Initially, there is a rise in the resting HR due to the increased physical activity; however, regular exercise without symptoms of overtraining leads to a decrease in the HR due to an increase in the parasympathetic activity and an optimisation of the cardiac output [133]. The expression of ion channels for the pacemaker potential is reduced [21]. Therefore, endurance training often results in exercise-induced bradycardia [134–137].	Initially, there is a fall in the HRV due to increased activity of the sympathetic system as a result of the physical activity [138], but regular physical activity leads to an increase in the parasympathetic activity which in turn causes a rise in HRV [36, 131, 138–140]. Endurance training normally increases the HRV [130, 131, 141–143]. These effects can be also seen in patients with myocardial infarction and patients with cardiac insufficiency [141] or Diabetes mellitus II [144].
Smoking	Active [145] and passive smoking [146] can lead to an increase in HR.	Smoking can lead to a decrease in HRV [147], this effect is dose dependent [146]. Even in non-smokers, passive smoking e.g. at home or at work leads to a reduction in the HRV [146–148].
Stress/mental tension	Stress (e.g. mental, workplace related) generally leads to an increase in the HR [149–152].	Stress (e.g. mental, workplace related) generally leads to decreased parasympathetic activity and thus to a reduction in the HRV [150, 152–157].
**External factors**		
Breathing	During inspiration there is a short-term increase in HR, during expiration there is a short-term decrease in HR [158, 159]. This is essentially due to pulmonary afferents from stretch receptors and interactions from central respiratory neurons to the circulatory center in the medulla oblongata.	The effects of respiration on HRV are reflected in the form of respiratory sinus arrhythmia (RSA) and is seen in the HF band. On the whole, the HRV parameter, RMSSD, does not seem to be affected by respiration [160]. For the rest of the parameters, the present state of knowledge is not conclusive [161–163].
Cold, low temperatures	In men, low ambient temperatures usually lead to a decrease in HR both at rest and during exercise, while in women there is no decrease in HR, but rather a slight increase in HR [164].	Only few studies about the effects of low temperatures on HRV are currently available: a reduction in the sympathetic activity and thus a raised HRV has been observed [165], while long-term exposure to cold, including in winter months or occupational exposure to cold, no influence on HRV could be shown [166–168].
Hazardous substances		Neurotoxic substances can lead to a reduction in the HRV: e.g. carbon disulphide [169, 170], however, not in the case of long-term low-dose exposure [171]; acute diesel and biodiesel inhalation [172]; chronic lead [173, 174], acute cadmium [175] or long-term mercury exposures [176] and neurotoxic styrene exposure [177, 178]. The data regarding the effects of chronic solvent exposure is not conclusive, both - a fall in the HRV and no differences - have been described [179–181]. In contrast, there was no evidence of a reduction in HRV through mercury exposure [182]. Only fetal mercury exposure appears to lead to a reduction in HRV [176]. Exposure to particulate matter (PM2.5) appears to reduce HRV [183].
Heat, high temperatures	High environmental temperatures lead to an increase in the HR [13, 184, 185] caused on the one hand by direct temperature effects on the sinoatrial node and on the other hand by the increase in sympathetic activity as a result of the activation of warm receptors.	High environmental temperatures lead to an increase in the sympathetic activity and a reduced HRV [166, 186].
Hypoxia		Hypobaric hypoxia usually leads to short-term sympathetic activation [187] and long-term to a reduction in HRV [188].
Noise	Noise often causes a rise in the HR [189], caused by activation of sympathetic nerves [190].	Only few studies that give information about the effects of noise on HRV are available; HRV appears to fall in the presence of noise [191–194].
Pharmacological drugs	Pharmacological drugs can have an increasing or decreasing effect on HR [195].	Pharmacological drugs can have an increasing or decreasing effect on HRV [195].
Shift work including night shift		Shift work with a night shift usually results in an activation of the SNS and a reduction in the PNS and thus a reduction in HRV, whereby there is a correlation between the duration of shift work in years and the reduction in HRV [196–202].
- the circadian rhythm should be kept in mind for comparable examinations.

### Quality assurance while determining the HRV

For quality assurance when determining HRV, it is recommended to take a resting ECG, check for artifacts and, if necessary, correct them, use recordings with only a few extrasystoles and choose a suitable analysis method and measurement duration, if possible, to use high sampling rates and to take possible influencing factors into account.

The following aspects should be taken into consideration for the purpose of quality assurance:

- a resting ECG should be recorded before the HRV is analysed in order to rule out cardiac arrhythmias (e.g. atrial fibrillation),
- recordings with more than 1% of ventricular or supraventricular extrasystoles should be evaluated critically because of the apparent increase in the HRV [203],
- checking for artifacts and, if possible, removal of artifacts (e.g. by visually checking the data during analysis, automatic methods for correcting artifacts),
- the analytical method of choice (e.g. Fast Fourier Transformation, Autoregressive Model, Trigonometric Regressive Spectral Analysis) to enable comparable interpretations (see Section: Analytical methods and parameters of HRV),
- the selected duration of recording (subsequent length of the sequence of analysis) or the underlying amount of data depending on the analytical method selected and the research question (see Appendix 1),
- a high sampling rate (see above),
- the possible influencing factors depending on the analytical method selected and the research question (see Table 2) and.
- the circadian rhythm should be considered as a possible confounder if comparing repeated measurements.

**Fig. 2:**
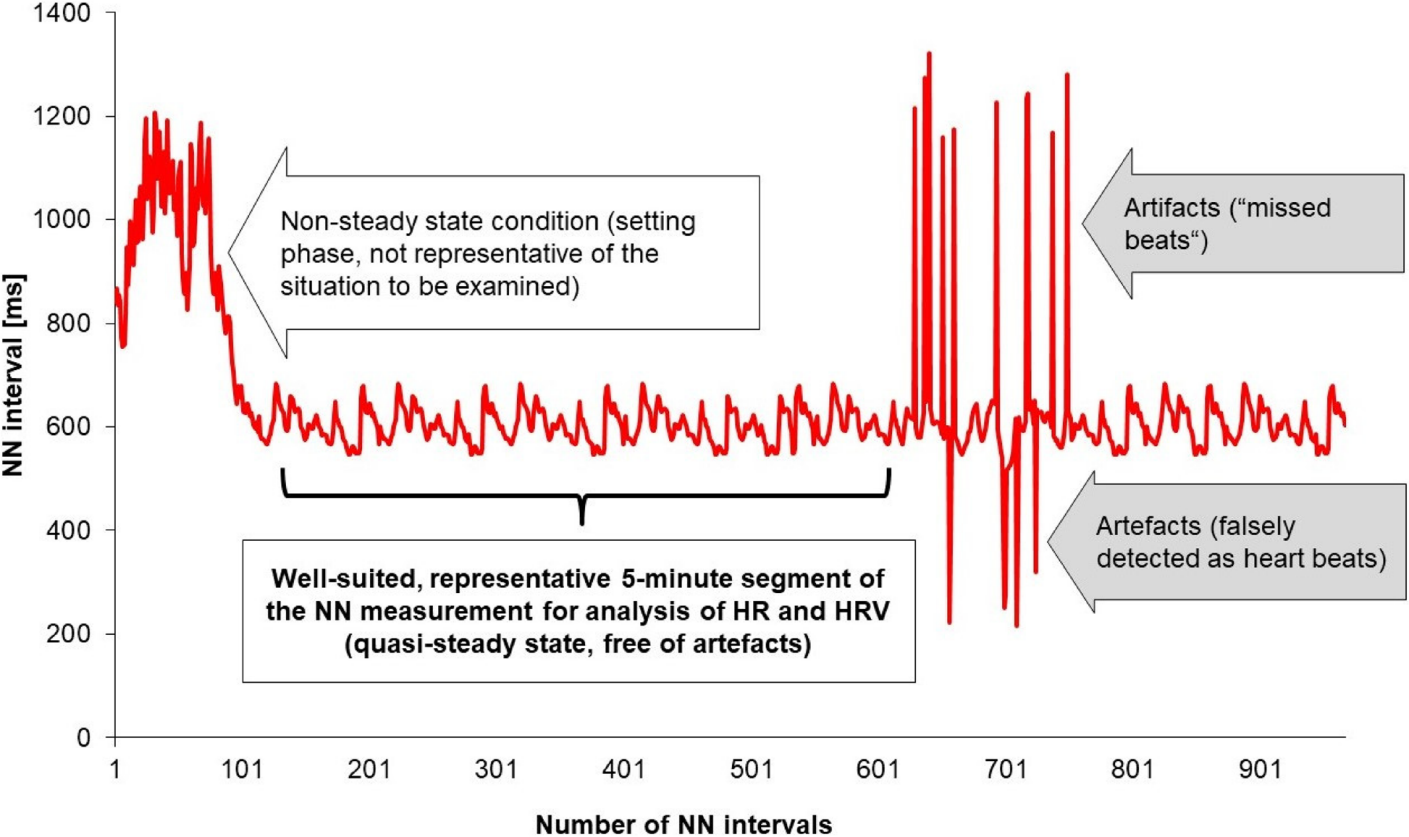
Principle of selecting a suitable 5-min range of the NN measurement from an artifact-superimposed recording with an early non-stationary range

In the case of short-term recordings, the selection of a suitable, representative area of the NN intervals is an important quality criterium for HRV analysis. For this, the non-steady setting phase at the beginning of the examination and the recordings with artifacts should be avoided for the analysis as far as possible (see Fig. 2).

### Other sources of interference

There are also other sources of interference (such as electromagnetic fields) that can influence the recording of HR and HRV.

When chest belts with wireless data transmission are used in the vicinity of electromagnetic fields from power poles or power supply lines [1] or used in vehicles and their vicinity [204, 205], interferences can occur. Artifacts due to body movements and due to electrical activity of other muscles can occur during physical activity. In the case of an ECG recording, these artifacts should be detected and manually removed at the end of the recording, whereas in cases of gathering data without ECG recording (like in most cases of chest belts systems), it is not always possible to attribute the artifacts to the movements.

## Analytical methods and parameters of HRV

HRV can be quantified using time and frequency domain methods as well as nonlinear analysis. Care must be taken to make the correct selection in relation to the objective and evaluation time.

**Fig. 3:**
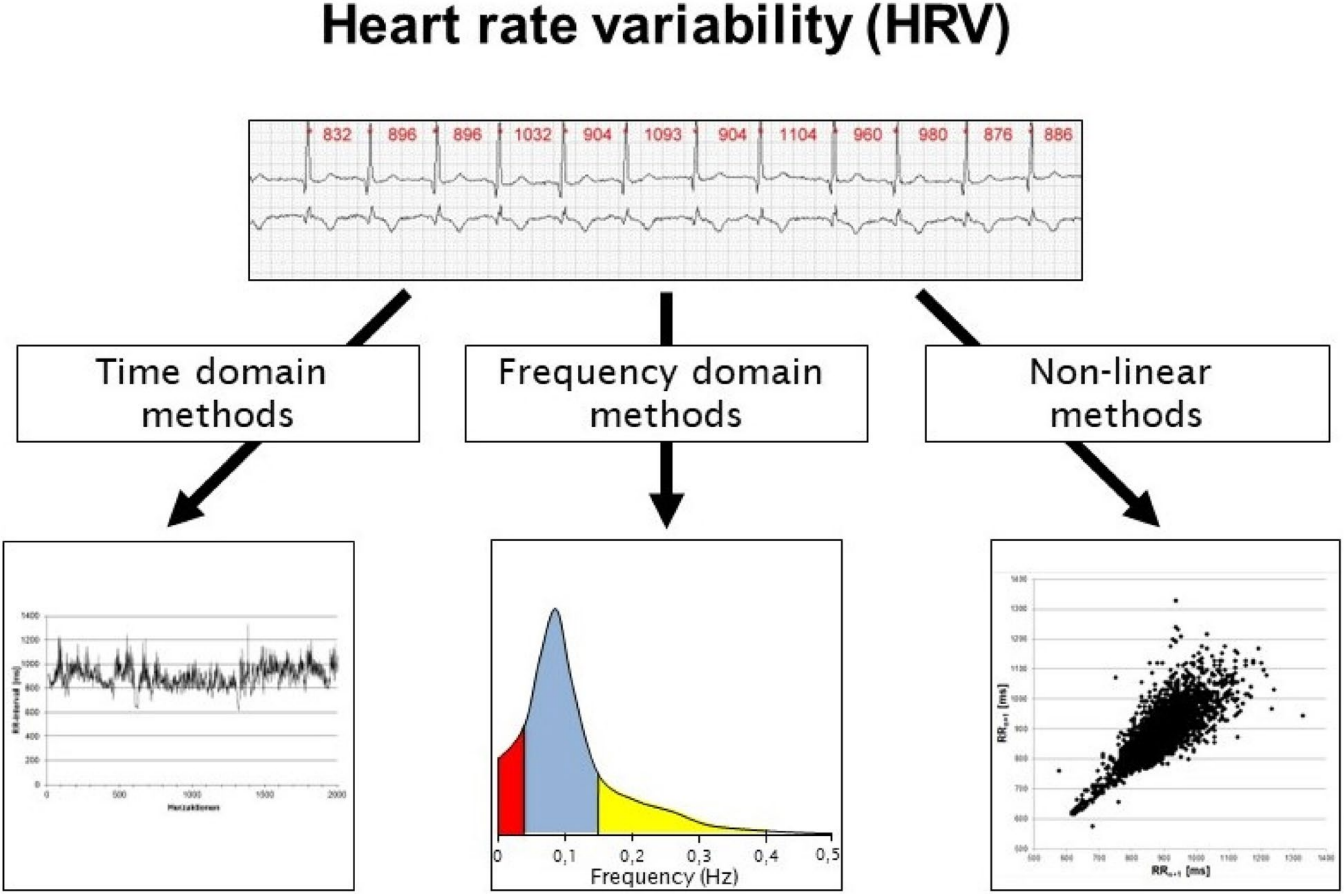
Overview of the possibilities of HRV analysis with examples of possible graphical representations

HRV is quantified using time and frequency domain methods as well as methods of non-linear analysis (see Fig. 3).

Time domain methods are divided into statistical and geometrical methods. In the case of the statistical methods, the NN intervals are evaluated mathematically with respect to its variance and the measurement of the rhythm is tagged with the time dimension or the percentage values, whereas geometrical methods provide an evaluation of HRV based on geometric forms. For these purpose histograms, HRV triangular index and its modifications, triangular interpolation of the NN interval histogram are used [206].

The analyses of the frequency range include, among others, the Fast Fourier transformation and Autoregression methods [207, 208]. Spectral analysis decomposes the periodic oscillation of the NN-signal, into different frequencies and amplitudes. This makes it possible to split the NN interval series and the periodic oscillations of the heartbeat into different frequencies and amplitudes, which in turn represent different physiological processes or different control systems [209].

In some cases, the Lomb algorithm is also used to analyse recordings with varying lengths and non-equidistant sampling. This represents a spectral analysis method of non-equidistantly sampled measured values [210]. The Lomb algorithm is an extremely slow method; but approximation methods have been established to speed up the application of the algorithm [211].

The methods of non-linear dynamics (e.g. Approximate Entropy [ApEn], Sample Entropy [SampEn], Detrended Fluctuation Analysis [DFA]) [30, 212–214] vary from the traditional time and frequency parameters in that they do not reflect the strength of the HRV, but they rather indicate qualitative aspects of the series of NN intervals [212]. These methods often prove to be suitable for long-term as well as short-term recordings and are considered more robust against artifacts.

One form of visualisation of the time series of NN intervals is offered by the so-called Poincaré Plot^5^ [213] (see Fig. 3). From this plot various indices can be determined and interpreted (e.g. length and width of the scatter-plot). Further, the form can also give hints about certain diseases [215].

A detailed listing of the HRV parameters is given in Appendix 1.

## Factors influencing the individual HR and HRV

HR and HRV are influenced by numerous changeable and non-changeable factors (uncontrollable physiological factors, diseases, controllable lifestyle factors and external factors), regardless of the acute stress.

Aside from acute physical exercise/exertion HR and HRV can be affected by several modifiable and non-modifiable factors. In addition to physiological parameters that cannot be influenced (e.g. age), there are a number of changeable influencing factors - e.g. living habits of the test subjects or the resulting consequences or external conditions. Furthermore, a variety of diseases are associated with reduced HRV, although the influence on the ANS can be viewed as a consequence of the disease and only rarely as a potential cause.

**Fig. 4:**
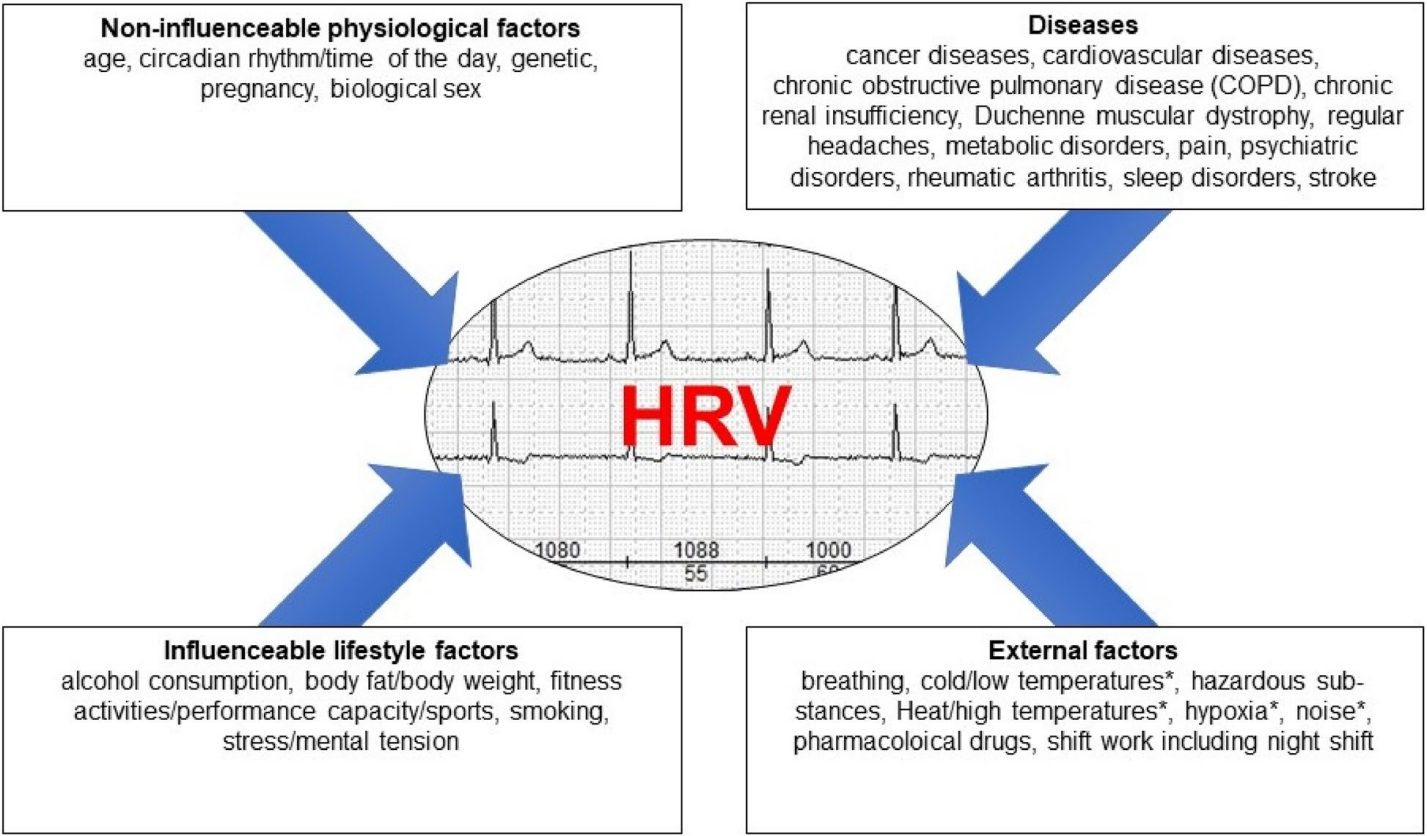
Grouping of the different factors influencing HRV into four main areas (modified taken from [216]).  * = HRV reduction as a result of the physiological response to the physical stimulus.

The individual influencing factors can be divided into four main areas (physiological factors that cannot be influenced, diseases, lifestyle factors that can be influenced and external factors) (see Fig. 4). The most relevant factors for investigations in the field of occupational medicine and occupational health science are described in Table 2. The consideration of these factors is of importance when HR and HRV are evaluated. In addition, various other factors and conditions (e.g. HRV in patients with sepsis that needs intensive care) have been mentioned in scientific literature. As these cases are normally not relevant in the field of occupational medicine and occupational health science they will not be considered any further in the current guideline.

Pharmacological drugs can have significant impact on the ANS or the electrical conduction system of the heart and thus should be considered when assessing and evaluating HR and HRV. Due to the large number of possible pharmacological interactions, the groups of beta blockers, acetylcholinesterase inhibitors, antiarrhythmics and psychotropic drugs are only mentioned here as examples [195].

## Evaluation and interpretation of HR and HRV

In order to be able to correctly evaluate and interpret the results of HR and HRV analyses, it is recommended that the specific question, the data collection method used and the evaluation strategy be coordinated in advance of the examinations.

For a valid and reliable evaluation and interpretation of HR and HRV adequate study designs, data sampling strategies and analysis methods are necessary prerequisites. HR and HRV parameters mirror the individual physiological workload response within a given context of individual, psychophysiological and work-related factors (see Table 2). Thus, measurements of HR and HRV should always be combined with complementary data (e.g., questionnaire about the subjective stress, perception of stress and the state of health). If possible, information about the ambient conditions at the workplace, like noise and temperature, should be collected at the same time.

### Heart rate (HR)

The main influencing factors for HR are dynamic loads on large muscle groups, but also static muscle loads, thermal and psychological loads.

Important factors that influence HR are dynamic activity of larger muscles, static muscular load of smaller muscles and thermal stress as well as mental workload [217, 218]. These factors often act together on the cardiovascular system and can induce a corresponding increase of HR during exertion. These effects were studied by e.g. Hettinger and Wobbe [189] in cases of different muscle, temperature and thermal radiation loads. A demarcation of the individual components is possible under controlled conditions only. So, HR during dynamic work of larger muscles can be used for estimating the energy expenditure only if the activity of smaller muscles and the mental workload are negligible and thermal conditions remain neutral [218]. Additional, static muscle strain as well as psychological and thermal strain lead to a decrease in the quality of the correlation between energy expenditure and HR (weak correlation coefficient).

### Heart rate at rest (HR_rest_)

It is recommended to use HR_rest_as the baseline value for interpreting HR changes under workloads

The HR_rest_ is the preferred baseline value for an individual evaluation of the HR during physical exertion (see Section: Quality assurance while determining the HR). Baseline measurement conditions (e.g. posture of the person, duration of recording) should be standardized to enable within- and between-subject comparisons. Both, an increased or a decreased HR_rest_ can be associated with an apparent cardiac disease [219, 220]. After considering physiological contributors to HR_rest_ (see Section: Factors influencing the individual HR and HRV) persons with unexplained higher (tachycardia) or a lower (bradycardia) HR_rest_ should be subjected to a cardiological examination.

Sometimes the determination of HR_rest_ is difficult in field studies, due to confounding effects on HR (psychological factors, environmental conditions like noise, ambient temperature etc.). Therefore, Hettinger and Wobbe [189] recommended the determination of a HR_reference_ during light dynamic work (e.g. 20 W on a bicycle ergometer for 10 min). Since this workload is typically perceived as a “light exertion”, the effect of psycho-emotional stress (“psychological heart rate“) is largely eliminated. Compared to the resting value in the supine position the HR increases by an average of 18.5 bpm in men during this procedure; while in women, an average increase of 24.5 bpm with relatively narrow limits of agreement can be expected [189].

### Maximum heart rate (HR_max_)

It is recommended to use the HR_max_as an exercise criterion and to determine it using a standardized exercise protocol.

The HR_max_ serves as a criterium for maximum physical exertion and can be determined during a standardised exhausting exercise protocol [221]. The most widely used methods for this are the treadmill and the bicycle ergometry. An optimal motivation to bring about the maximum performance and the observance of the stop criteria are the main requirements for the determination of the HR_max_. Personal physical exertion can also be measured using the Borg scale as an example [222, 223]. However, one should keep in mind that apart from factors like age, sex and fitness level [224] and certain bradycardia producing drugs [8], the value of the HR_max_ determined largely depends on the muscle mass that is used.

For an appropriate estimation of cardiac workload, the interpretation of the HR response during a given (occupational) physical task should always be referred to the individual HR_rest_ and HR_max_ (see Section: Physiological mechanisms). Here, a value of the HR during physical exertion (occupational), which lies closer to the HR_max_, indicates a higher degree of stress on the heart. The continuous performance limit can also be referred to for the interpretation (see Section: Continuous performance limit (CPL)).

### Recovery heart rate (HR_recovery_)

HR_recovery_can be used to estimate the recovery ability of the cardiovascular and metabolic systems.

The HR_recovery_ can be used to estimate the recovery capacity of the cardiovascular and the metabolic systems. It strongly correlates with the function of the parasympathetic branch of the ANS [225] and typically decreases exponentially after the end of the exertion. The main factors that influence the temporal kinetic of the recovery of the vagus are intensity, duration and method of the physical exertion, initial performance level and the type of recovery [226–228].

### Continuous performance limit (CPL)

The CPL for physical work is the maximum physical work that can be maintained over a work shift (approx. 8 h) without progressive symptoms of fatigue.

The CPL of physical exertion characterizes the maximum muscular work that can be maintained over a regular working shift (about 8 h) without any progressive symptoms of fatigue and where the measurable physiological parameters return to baseline or fall even below baseline within 15 min after the work cessation [13]. If the CPL is adhered to, overloading and injuries can be avoided and adequate recovery for the next (work) shift is possible. The CPL can be used for the identification of muscular physical exertion without fatigue (below the CPL) and muscular exertion inducing fatigue (above the CPL) with respect to an 8-hour working shift [10, 229, 230]. The value of CPL can be determined using cardiac (e.g., HR) as well as metabolic parameters (e.g., energy turnover, lactate). Spiroergometry can also be used as an alternative method for the determination (e.g. 40% of the maximum oxygen intake). HR in particular is suitable as an easy-to-collect cardiac parameter for recording cardio-pulmonary stress. In the cases of dynamic activity of larger muscle masses, the CPL ranges between 105 and 110 bpm or alternatively between HR_rest_ + 30–35 bpm [13]. It should be noted that HR used for the determination of the CPL, like the individual HR_rest_ and HR_max,_ also underlies a strong individuality due to e.g., age and the level of physical fitness.

**Fig. 5:**
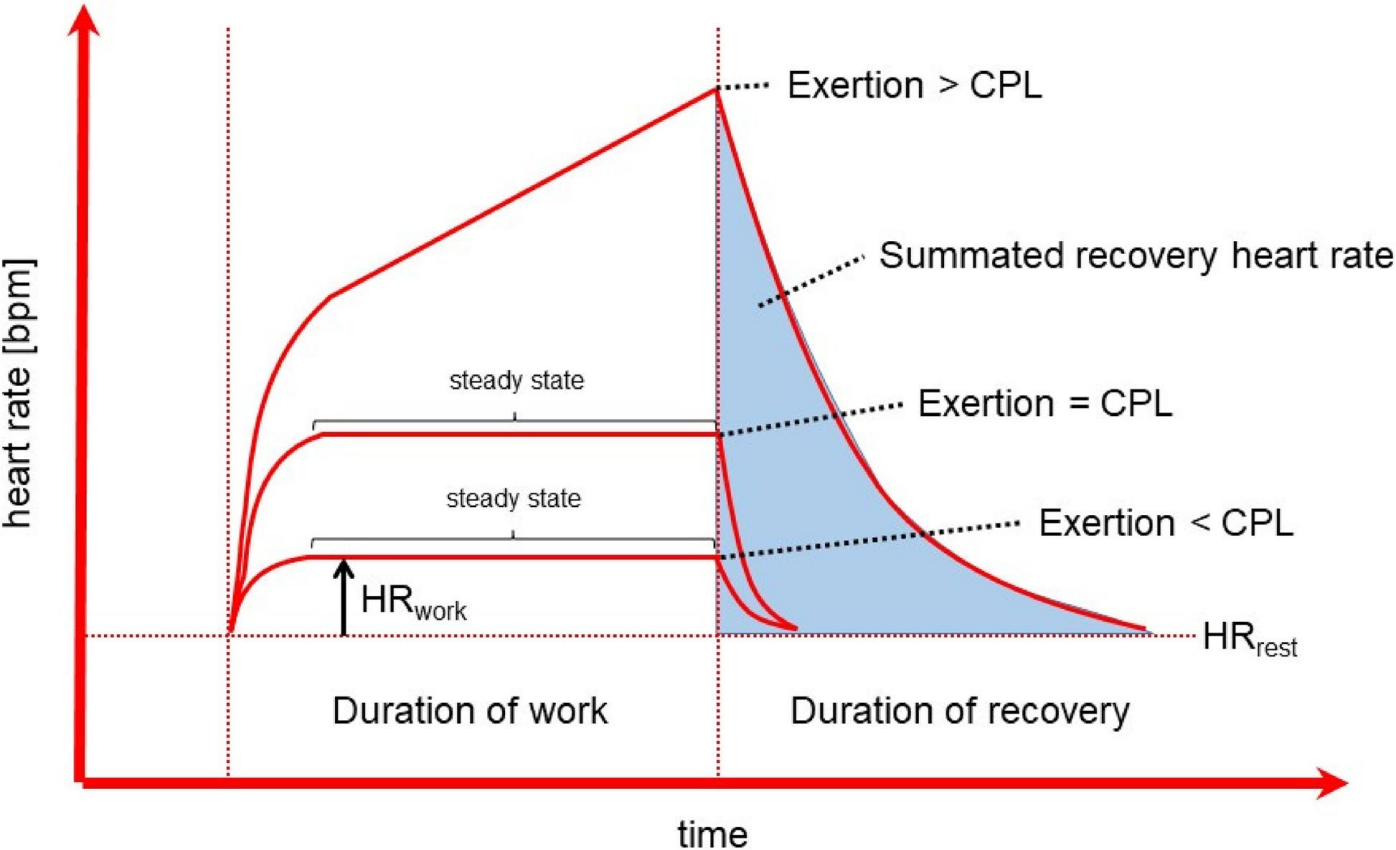
Heartrate during different loads (below or above the CPL as well as in the range of the CPL) with respective recoveries, schematic representation, modified according to Müller [14], CPL = continuous performance limit

Below the CPL, the HR shows a linear increase along with the intensity of workload. In the case of light work with a constant performance over time, the HR reaches an almost constant deflection (“steady state“) within a short period of time (few minutes). Typically, this “steady state“ can be maintained over the entire 8-hour working shift (see Fig. 5).

Small, short-term overshoots beyond the CPL (e.g., HR of 130–140 bpm) are common during a work shift and do not pose any health risks, while scheduled breaks during constant physical exertions with a HR > 130 bpm help to overcome muscular fatigue. The more intense the physical strain is and the more the state of exhaustion is reached, i.e., the further the CPL is exceeded, the more these tiring activities must be limited with respect to duration.

If the CPL is being continuously exceeded, this kind of work is classified as heavy physical work or hard labour, in terms of energy [231]. It leads to increasing muscle fatigue (along with anaerobic metabolism), which is generally reversible without any effects on health. There is a continuous rise in HR and a rise in fatigue as well (see Fig. 5). Heavy physical work is also relevant from the motor biomechanical point of view, because the skeletal system (joints, intervertebral discs) might be injured under relevant conditions [230]. However, these aspects are beyond the scope of this guideline.

Apart from the CPL, the integral of HR_recovery_ (see Fig. 5) is also considered an indicator of the individual physical exertion [14].

It should be noted that an evaluation of workload based on the CPL (as described above) is only valid, if larger muscle masses (> 1/6 of the total muscle mass) are dynamically active. If smaller muscles masses are used for dynamic tasks, the CPL has to be adjusted (e.g., decreased) in proportion to the muscle mass used. Typically, HR, HR_work_ and oxygen update are lower compared to whole body exertion, despite similar or even shorter time until exhaustion. Sometimes, e.g. in cases of work done by the arm or hand, the CPL is not valid for estimating time to fatigue or exhaustion [232]. Under these conditions local muscle load is limiting for endurance of work. In cases of isometric muscular work or with an increasing portion of isometric muscle activity during dynamic tasks, an evaluation of the workload intensity with respect to the CPL is not valid.

With respect to work structuring, the occupational tasks that are seen as being causal for an increase over the CPL should be considered in detail.

### Heart rate variability (HRV)

In order to interpret HRV parameters under external stress, it is recommended that sensible planning and implementation of the measurement of cardiac actions be taken into account, especially with regard to the length of the recording.

Several methods are available for the analysis of HRV. In principle, the time domain parameters of HRV can be calculated using mathematical functions in established spreadsheet programmes. In addition, many manufacturers of mobile ECG instruments provide software programmes that enable the calculation of the time domain and the frequency domain parameters of HRV and also permit a non-linear analysis of the NN interval series. Freely available software packets with good documentation are also available.

For statements about the ANS (see Sections: Application to assess physical exertion and Application to assess mental workload), suitable HRV parameters should be used and recorded or analyzed with a time range suitable for the activity or rest measurement (see Appendix 1). It must be taken into account here whether an influence of the SNS or PNS is to be measured primarily and how long the recording time can reasonably be carried extended.

While assumptions based on specific HRV parameters require long-term recordings (see Appendix 1), or the 24-hour measurement of HRV can be advantageous for gaining an overall impression of the ANS, HRV parameters that are suitable for short-term measurements are primarily used for the evaluation of physical stress at work with rapidly changing requirements. The first minute after a change in load should not be included in the data analysis, because transient adaptation processes of the vegetative control circuits dominate during this phase.

Due to the high inter-individual variability and numerous exogenous and endogenous factors affecting the NN interval, which usually cannot be changed as part of a measurement of the NN intervals, HRV analysis should be used only in combination with a baseline assessment or with repeated measurements during work under the comparable conditions.

Some studies with reference values are available for the comparability of resting measurements. Attention must be paid to the age and biological sex dependence of HRV and to a reference population fitting to the test person.

In recent years, a number of studies has been published that use reference values to differentiate between increased and reduced HRV in individual HRV measurements. Due to the age and biological sex dependence of HRV described above, corresponding HRV reference values should also take this into account, but this has only been done in some of the published studies. An overview of studies with reference values can be found in Appendix 2. Furthermore, due to the possible genetic influence of ethnicity, a study should be used which was composed of test subjects who are similar to the working population.

Even though this provides limit values for HRV parameters, it should be borne in mind that these were calculated purely empirically. Therefore, no general health-related statements are possible. Standardised serial measurements (individual longitudinal studies) of HRV in combination with the medical history, clinical examination and other methods (e.g. questionnaires) can be valuable in explaining the individual health risks and help to evaluate the effectiveness of medical preventive measures.

## Application in the fields of occupational medicine and occupational health science

In the field of occupational medicine and occupational health science, HR and HRV can be used to answer various questions.

The methods used for the recording and evaluation of HR and HRV can be used to gain an objective view of the activity of the ANS. The applications in the fields of occupational medicine and occupational health science may be summarized:

- complementary examinations for the risk analysis and risk assessment to identify the core areas of work-related stress,
- analysis of the individual physical and mental workload and a process-integrated measurement for an objective view of the workload response over the course of the working day,
- determination of a health status indicator,
- derivation of actions to be recommended for each individual e.g. workplace design,
- determination of the fatigue and recovery behaviour and.
- evaluation of interventions in medicine and occupational medicine.

### Application to assess physical exertion

The use of HRV during physical stress, especially during dynamic muscle work, has long been established. The analysis of HRV also offers added value compared to the linear behaviour of HR due to the often two-phase or multi-phase behaviour under increasing loads.

The HRV parameters SDNN, RMSSD, Total Power, LF-Power and/or HF-Power should be used for this purpose. A minimum recording time of 5 min is recommended

The evaluation of physical exertion using the HR especially during dynamic muscle work has been established for a long time. The knowledge gained through HRV in such cases and under standardised conditions are: (i) a proven correlation between HRV parameters and (ii) the metabolic and respiratory stress indicators, (iii) the multi-phase course during progressively increasing exertion and (iv) the recovery behaviour after varying degrees of exertion [233, 234]. This enables an accurate evaluation of the physical exertion without the use of a time-consuming, cost-intensive recording method that is also partly unavailable in the ambulatory and reactive forms. In addition to the parameters of total variability, e.g. SDNN or Total Power, the HRV parameters RMSSD, LF power and HF power and the non-linear indices are suitable for the determination of the acute physical exertion. These often show a two-phase or multi-phase behaviour under increasing load and, thus, have an added value compared to the linear behaviour of the HR, especially under moderate and high physical loads [235, 236].

### Application to assess mental workload

HR and HRV can be used for mental stress assessment. However, the selection of suitable HRV parameters is limited

The HRV parameters RMSSD, LF, HF, LF/HF, DQ and SD1 should be used for this purpose. A minimum recording time of 5 min is recommended

Mental stress is characterised by deflections in HR and HRV, which can therefore be used as mental stress indicators. Since the construct of mental workload is difficult to measure, HR and HRV are taken as parameters of general activation and can be used to reflect the vegetative balance of the organism. These parameters may therefore be used to draw conclusions about mental stress [101, 237–248]. In addition, HRV can also be used as an indicator of both - the psychophysical condition of the organism and the restriction in the adaptability for biopsychosocial problems. The HRV parameters, RMSSD, LF, HF or LFnu and HFnu, LF/HF as well as DQ and SD1 are considered mental workload indicators. However, ULF and VLF are not suitable. Resting HRV may not be a predictor of cognitive capacity in cross-sectional studies.

### Application for risk stratification of diseases

A reduced HRV correlates with increased morbidity and mortality in some diseases (e.g. after myocardial infarction, coronary bypass surgery, heart failure, stroke, chronic obstructive pulmonary disease, and high blood pressure)

A prognostic value of HRV has currently only been proven for a few diseases. Large cohort studies have shown, among other things, that mortality was significantly higher in patients after myocardial infarction with a lower HRV compared to patients with higher HRV parameters in the post-infarction phase [77, 249, 250]. According to a meta-analysis of five studies with a total of 3,489 patients, the mortality risk in patients after myocardial infarction and an SDNN < 70 ms was 21.7%, while for n SDNN > 70 ms it was 8.1% [249]. The threeyears mortality rate after myocardial infarction was thus 2–3 times higher in the group with low HRV.

A correlation between reduced HRV (here SDNN < 93 ms) and recurrence of a coronary event was also found in patients after coronary bypass surgery. During an average three-year follow-up period, 13 out of 74 patients with reduced HRV suffered such an event, while this only affected 3 out of 132 patients in the comparison group (SDNN ≥ 93 ms) [251]. A correlation between low HRV and overall mortality or cardiac endpoints was also found for patients with heart failure [252] and in patients with strokes, the individual HRV after the stroke correlated with the long-term outcome [122].

Schmidt et al. [253] were able to show in intensive care patients with multiple organ failure that the logarithmic value of the frequency-related HRV parameter VLF (lnVLF) allows an estimate of mortality in the short-term prognosis (up to 60 days). A systematic review of the relationship between chronic obstructive pulmonary disease and HRV reported that the reduction in HRV correlates with the severity of COPD [78]. Furthermore, there appears to be a correlation between a higher HRV and longer survival in the context of tumor disease [254, 255]. In addition, it has been shown that HRV is reduced in individuals diagnosed with type I diabetes mellitus even before clinical signs of autonomic dysfunction appear [256].

There also appears to exist a correlation between a lower HRV and later manifestation of hy-pertension. In a group of 2,061 subjects controlled for age, biological sex, ethnicity, current smoking status, diabetes mellitus and educational status, subjects in the lowest quartile of HRV showed a 2.44-fold increased risk of new manifestations of hypertension after three years [257]. Based on data from the Framingham Heart Study (2,024 subjects), Singh et al. [258] were able to show an increased risk of developing hypertension with reduced HRV in men, but not in women. Schroeder et al. [259] studying 11,061 subjects, found in those individuals which belonged to the lowest quartile - in relation to the RMSSD value - a 1.36 higher risk of developing high blood pressure compared to individuals of the highest quartile.

Reduced HRV correlates with prognosis scores for the occurrence of cardiovascular events

A significant correlation between the reduction of the HRV parameter RMSSD and various prognosis scores for the occurrence of coronary heart disease or stroke was demonstrated in a collective of 11,994 subjects from the Mannheim industrial cohort [260]. This could be shown for the PROCAM score according to Assmann et al. [261], for the Coronary Heart Disease Framingham Score [262] and for the cardiovascular prognosis indicators SCORE, related to the risk of coronary heart disease (SCORE-CHD) and cardiovascular disease (SCORE-CVD) according to Conroy et al. [263].

### Application to evaluate preventive measures

HRV can be used as part of the evaluation of prevention measures in compliance with the quality criteria. Repeated HRV measurements should be used for this purpose

HRV has been established particularly as a useful parameter for the evaluation of preventive measures like stress reduction courses, dietary changes, judicious use of stimulants, changes in eating behaviour, sport activities including the preventive monitoring of overtraining syndromes [264, 265] and measures to reduce weight in order to evaluate the success of the corresponding preventive or interventional measures in longitudinal comparisons [266]. For example, a change in the sympathetic-parasympathetic balance and a higher parasympathetic baseline activity (e.g. raised SDNN or RMSSD, reduced LF/HF ratio) indicate efficacy of the preventive measures.

### Application in biofeedback

HR and HRV parameters can be used to objectify relaxation effects in the context of biofeedback. Long-term effects could not be shown with HRV-based biofeedback methods

HR and HRV have been used for biofeedback in cases of stress recovery and recently also in the treatment of posttraumatic stress disorder e.g. for an objective view on the effects of stress relaxation [156, 267–270]. However, until now, only short-term effects of such interventions have been observed. It has not yet been possible to demonstrate a long-term effect [270]. In addition, suitable and validated methods must be used to utilise the short-term effects of HRV biofeedback methods.

With reference to the determination and the evaluation of HRV (see Chap. 4), it is inevitable that the biofeedback methods, which determine the HRV with the help of pulsoximeter or respiratory activity, cannot be seen as valid measurement methods to assess HRV and therefore cannot be recommended for HRV-based biofeedback.

## Conclusions

The practicability of the HR and HRV analysis on a daily basis for field studies at workplaces has been proven. These analytical methods can be used with a goal-oriented approach for various tasks when the methodological requirements are met. Under these conditions, HR and HRV can be recommended for the use not only in research institutes, but also for practising by occupational physicians and company doctors. This might help to improve diagnostic efficiency and to elucidate heart and health related mechanisms in the field of modern occupational medicine facing an ever-changing working environment and a demographic change in general. For a practical use a checklist is attached in the appendix to support scientists and users.

### Supplementary Information

**Supplementary Material 1.**

## Data Availability

No datasets were generated or analysed during the current study.

## Appendix 1: parameters of HRV

**Table:**

Method	Measure of variability	Other names	Unit of measurement	Definition and explanation	Indicator of …	Activity as part of the autonomic nervous system	Recommendation for evaluation time
Time domain methods							
Statistical	SDNN	RRSD, SD, SDRR	ms	Standard deviation of NN intervals within the measurement area	Short-term and long-term variability [43]	Sympathetic and parasympathetic [44]	
	CVRR	CV	n.o.	Coefficient of variation of NN intervals, equal to the standard deviation of NN intervals divided by the mean of NN intervals	No clear assignment	No clear assignment	
	SDANN		ms	Standard deviation of the average of all consecutive 5-minute NN intervals for estimation of HRV for long-term measurements	Long-term variability [44]	No clear assignment	Long-term recording, ideally 24 h
	RMSSD	R-MSSD, rMSSD	ms	Root Mean Square of successive differences: Square root of the arithmetic mean of the squared differences between adjacent NN intervals	Short-term variability [43]	Parasympathetic	
	SDNN-Index	SDANN-Index, SDNN_i_	ms	Standard deviation of the average of all normal NN intervals of 5-min segments from the 24-hour ECG	Long-term variability, short-term variability [43]	No clear assignment	Long-term recording, ideally 24 h
	NN 50		n.o.	The number of pairs of neighbouring NN intervals that deviate from one another by more than 50 ms	Spontaneous variability, long-term variability	Parasympathetic [43]	
	pNN 50		%	Percentage of consecutive NN intervals that deviate from one another by more than 50 ms	Spontaneous variability, long-term variability	Parasympathetic [44]	
	SAa		ms	Absolute sinus arrhythmia: sum of the differences of the consecutive NN intervals, divided by their number	No clear assignment	No clear assignment	
geometric	HRV triangular Index	RR triangular index	n.o.	The integral of the density distribution (number of all NN intervals divided by the maximum (height) of the density distribution) or ratio of the absolute number of all NN intervals to the number of all modal NN intervals	Total variability	No clear assignment	At least 20 min
	TINN		ms	Triangular interpolation of NN interval histogram: is the baseline width of the minimum square difference of the triangular interpolation for the highest value of the histogram of all the NN intervals	No clear assignment	No clear assignment	At least 20 min
**Frequency domain methods**							
FFT (Fast Fourier Transformation) and Autoregressive Model (AR)	TP		ms^2^	Total power: total performance or total spectrum; corresponds to energy density between 0.00001 to 0.4 Hz	Total variability	No clear assignment	
	ULF		ms^2^	Ultra very low frequency: power density spectrum below 0.003 Hz	No clear assignment	No clear assignment	
	ULF%		%	Percentage of ULF in the total spectrum	No clear assignment	No clear assignment	
	VLF		ms^2^	Very low frequency power: power density spectrum in the frequency range of 0.003 to 0.04 Hz	No clear assignment	Parasympathetic [45]	
	VLF%		%	Percentage of VLF in the total spectrum	No clear assignment	Parasympathetic [45]	
	LF	B Band	ms^2^	Low frequency power: power density spectrum in the frequency range of 0.04 to 0.15 Hz	No clear assignment	Sympathetic and parasympathetic	At least 5 min [46]
	LF%	relative B Band	%	Percentage of LF in the total spectrum	No clear assignment	Sympathetic and parasympathetic	At least 5 min [46]
	HF	C Band, respiratory sinus arrhythmia, Respiratory	ms^2^	High frequency power: power density spectrum in the frequency range of 0.15 to 0.40 Hz	No clear assignment	Parasympathetic [47]	At least 5 min [46]
	HF%	C Band, respiratory sinus arrhythmia, Respiratory band	%	Percentage of HF in the total spectrum	No clear assignment	Parasympathetic	At least 5 min [46]
	LF nu	LF n.U., LF norm	nu	Low frequency normalised unit: standardized power or power of the LF in standardized units; corresponds with LF/(TP-VLF) x 100^1^	No clear assignment	Sympathetic and parasympathetic	At least 5 min [46]
	HF nu	HF n.U., HF norm	nu	High frequency normalized unit: standardized power or power of the HF in standardized units; corresponds with HF/(Total Power – VLF) x 100^1^	No clear assignment	Parasympathetic [47]	At least 5 min [46]
	LF/HF	Quotient of LF and HF; LF/HF Ratio	n.o.	Quotient of the spectrum in LF and the spectrum in HF	No clear assignment	Ratio or coefficient or ratio between LF and HF band power [47]	At least 5 min [46]
	VLF Peak		Hz	Very low frequency peak: frequency peak in the VLF band; thermoregulation peak	No clear assignment	No clear assignment	
	LF Peak		Hz	Low frequency peak: frequency peak in the LF band; baroreflex peak	No clear assignment	No clear assignment	At least 5 min [46]
	HF Peak		Hz	High frequency peak: frequency peak HF band; respiratory peak	No clear assignment	No clear assignment	At least 5 min [46]
**Non-linear methods**							
Poincaré-Plot	DL	D_L,_ Lorenz length	ms	Length of the major axis of the ellipse (95% confidence region)	Long-term variability	No clear assignment	
	DQ	D_q_, D_W_, Lorenz width	ms	Length of the minor axis of the ellipse (95% confidence region)	Short-term variability	No clear assignment	
	SD1	SDQ, SDw, stdb, SO_Q_, SD-horizontal, SO_W_	ms	Standard deviation of the distances of the points from the minor axis^2^	Short-term variability [40, 48]	Parasympathetic [49]	
	SD2	SDL, SD-vertical, stda, SO_L_	ms	Standard deviation of the distances of the points from the major axis^2^	Long-term variability [40, 48]	Sympathetic and parasympathetic [49]	
Detrended fluctuation analysis (DFA) or trend-correcting fluctuation analysis	DFA1	alpha 1	n.o.	The degree of coincidence /correlation; ranges from 0.5 (coincidental) to 1.5 (correlated) with normal value of 1.0; is often used as a non-linear parameter for short NN interval data	Short-term variability [40, 50, 51]	No clear assignment	
	DFA2	alpha 2	n.o.	Is often used as a non-linear parameter for RR intervals of longer durations of recording, reduced values are associated with a bad prognosis	Long-term variability [40, 50, 51]	No clear assignment	
	D2		n.o.	Correlation dimension	No clear assignment	No clear assignment	
Recurrence plot	Lmean		beats	Mean line length	No clear assignment	No clear assignment	
	Lmax		beats	Max line length	No clear assignment	No clear assignment	
	REC		%	Recurrence rate	No clear assignment	No clear assignment	
	DET		%	Determinism	No clear assignment	No clear assignment	
	ShanEn		beats	Shannon Entropy	No clear assignment	No clear assignment	
Others	ApEn		n.o.	Approximate entropy, High values correspond to high variability, values independent of the recording length [52]	Short-term variability [40]	No clear assignment	
	SampEn		n.o.	Sample entropy: High values correspond to high variability, values independent of the recording length [52]	Short-term variability [40]	No clear assignment	

*n.o* no unit assigned.

^1^ LF nu and HF nu behave reciprocally to each other, the sum of both parameters equals 100%.

^2^ SD1 and SD2 correlate directly with the HRV parameters SDNN and the SDNN index and are therefore not classified by some authors as non-linear parameters, but rather as time-related HRV parameters [53].

## Appendix 2: overview of the various studies with details of the standardised values

**Table:**

Study	Probands/ age group	*N*	Duration of HRV-analysis	Age-related	Biological sex-related	Specification of the standard values as…
Bigger et al. 1995 [245]/ ESC/NASPE 1996 [47]	adults	274	24 h	no	no	MV ± SD
ESC/NASPE 1996 [47]	adults	n/a	5 min	no	no	MV ± SD
Nunan et al. 2010 [246]	systematic review	1–36 studies	5 min	no	yes	MV ± SD
Kim & Woo 2011 [247]	adult participants in check-up examinations in Korea	3,396	5 min	yes	yes	MV ± SD
Voss et al. 2012 [81]	representative adult population from Germany	1,906	5 min	yes	no	percentile
Zeng et al. 2014 [248]	probands from China	371	15 min	yes	yes, but no difference detected	percentile
Voss et al. 2015 [242]	representative adult population from Germany	1,906	5 min	yes	yes	MV ± SD
Sammito & Böckelmann 2017 [243]	adult population from Germany (20–60 years)	695	24 h	yes	yes	percentile
Sammito & Böckelmann 2017 [244, 244]	adult population from Germany (20–60 years)	673	5 min	yes	yes	percentile

*MV *Mean value, *SD *Standard deviation, *n/a *not available

## Abbreviations

ANS: Autonomic nervous system
ApEn: Approximate Entropy
BMI: Body weight index
bpm: beats/min
COPD: Chronic obstructive pulmonary disease
CPL: Continuous performance limit
DFA: Detrended Fluctuation Analysis
HIIT: High-intensity interval training
HR: Heart rate
HR_max_: Maximum heart rate
HR_net_: Net heart rate
HR_recovery_: Recovery heart rate
HR_reference_: Reference heart rate
HR_rest_: Resting HR
HRV: Heart rate variability
HR_work_: Working heart rate
MV: Mean value
n.o: No unit assigned
n/a: Not available
PNS: Parasympathetic nervous system
RSA: Respiratory Sinus Arrhythmia
SampEn: Sample Entropy
SD: Standard deviation
SNS: Sympathetic nervous system

## Footnotes

1 NN interval = normal-to-normal interval, used synonymously with the terms RR interval, IBI (interbeat interval), cycle length variability, heart period variability.

2 In some load protocols, the HR is also analysed after 3–5 min after the end of the load.

3 Older publications often use the term work pulse rather than work HR.

4 The following procedure is recommended: The measurement should preferably be taken in a sitting position, after at least five, ideally 15 min of rest and should be determined by two consecutive measurements. The measurement duration should be at least 30 s. The person should have avoided smoking, eating, caffeine or physical exertion half an hour before the measurement [28, 29].

5 The terms, Lorenz plot or Scatter plot, are used as synonyms.
