# Supplementary material for: Guideline for the application of heart rate and heart rate variability in occupational medicine and occupational health science
Source: J Occup Med Toxicol. 2024 May 13;19:15. doi: 10.1186/s12995-024-00414-9 (PMC11089808; doi:10.1186/s12995-024-00414-9)
Supplement: Supplementary file 1 — Supplementary Material 1. [file 12995_2024_414_MOESM1_ESM.docx]

**Checklist**

| **Define the research question(s)** | |
| --- | --- |
| - How can HR/HRV measures can help you answer these research questions? | 🡪 see section 7 |
| - Identify the covariates and confounders (endogenous and exogenous factors) that may also affect HR/HRV in your study | 🡪 see section 6 (Table 3) |
| **Measurements** | |
| - Choose appropriate measurement systems/tools | 🡪 see section 4.1 (Table 1) |
| - Select proper leads and electrodes, and modes of skin preparation | 🡪 see sections 4.2 – 4.4 |
| **Minimizing Error** | |
| - Quality assurance when determining the HR and HRV | 🡪 see sections 4.5 and 4.6 |
| - Avoiding potential signal interference and artefact | 🡪 see section 4.7 |
| **Analyses** | |
| - Choose appropriate analytic methods and parameters | 🡪 see section 5 |
